# Elasticity of Ferropericlase across the Spin Crossover in the Earth’s Lower Mantle

**DOI:** 10.1038/srep17188

**Published:** 2015-12-01

**Authors:** Jing Yang, Xinyue Tong, Jung-Fu Lin, Takuo Okuchi, Naotaka Tomioka

**Affiliations:** 1 Department of Geological Sciences, Jackson School of Geosciences, The University of Texas at Austin, Austin, TX 78712, USA; 2 Center for High Pressure Science and Technology Advanced Research (HPSTAR); 3 Institute for Study of the Earth’s Interior, 827 Yamada, Misasa, Tottori, 682-0193 Japan

## Abstract

Knowing the elasticity of ferropericlase across the spin transition can help explain seismic and mineralogical models of the lower-mantle including the origin of seismic heterogeneities in the middle to lowermost parts of the lower mantle^1^,^2^,^3^,^4^. However, the effects of spin transition on full elastic constants of ferropericlase remain experimentally controversial due to technical challenges in directly measuring sound velocities under lower-mantle conditions^1^,^2^,^3^,^4^,^5^. Here we have reliably measured both *V*_*P*_ and *V*_*S*_ of a single-crystal ferropericlase ((Mg_0.92_,Fe_0.08_)O) using complementary Brillouin Light Scattering and Impulsive Stimulated Light Scattering coupled with a diamond anvil cell up to 96 GPa. The derived elastic constants show drastically softened *C*_*11*_ and *C*_*12*_ within the spin transition at 40–60 GPa while *C*_*44*_ is not affected. The spin transition is associated with a significant reduction of the aggregate *V*_*P*_*/V*_*S*_ via the aggregate *V*_*P*_ softening because V_*S*_ softening does not visibly occur within the transition. Based on thermoelastic modelling along an expected geotherm, the spin crossover in ferropericlase can contribute to 2% reduction in *V*_*P*_*/V*_*S*_ in a pyrolite mineralogical model in mid lower-mantle. Our results imply that the middle to lowermost parts of the lower-mantle would exhibit enhanced seismic heterogeneities due to the occurrence of the mixed-spin and low-spin ferropericlase.

Seismic wave studies of the lower mantle have established relatively reliable seismic models including compressional and shear wave velocities (*V*_*P*_ and *V*_*S*_) in one-, two-, and three-dimensional tomographic models (e.g., PREM, AK135, S40RTS)^6^,^7^,^8^. Thus far, it has been shown that most of the lower mantle, except the lowermost mantle such as the D′′ layer, exhibit relatively smooth changes in seismic parameters that have been commonly interpreted as a result of the high pressure-temperature (*P-T*) effects on physical properties of candidate minerals in the region. The lower mantle is mostly believed to be seismically and chemically homogeneous and likely consisting of approximately 75% bridgmanite (silicate perovskite (Mg,Fe)(Al,Fe,Si)O_3_; Pv), 20% ferropericlase ((Mg,Fe)O; Fp), and 5% calcium perovskite (CaSiO_3_) by volume in a pyrolite compositional model^9^,^10^. However, a number of seismic studies have shown that possible thermal and/or chemical heterogeneities, especially in the middle to lower part of the lower mantle ranging from approximately 1500 km to 2800 km in depth, are needed to reconcile differences between our current understanding of seismic models and mineral physics results^11^,^12^,^13^,^14^,^15^. Other than the bridgmanite to post-perovskite structural transition at the D′′ zone region^16^, which may be responsible for the seismic discontinuities in the lowermost mantle, the electronic spin transitions of iron in lower-mantle minerals have been suggested to affect our understanding of mid to lowermost lower-mantle seismic heterogeneities.

The electronic spin transition of iron in lower-mantle bridgmanite and ferropericlase has been recently reported to affect physical and chemical properties of the host minerals, including changes in elasticity, iron partitioning, and electrical and thermal conductivities^1^,^4^,^17^,^18^,^19^,^20^,^21^,^22^, that may contribute to seismic heterogeneities of the region. It has been shown that a broad spin crossover occurs in ferropericlase at conditions ranging from 1700 km to 2700 km in depth^23^, while the Fe^3+^ in the octahedral site of bridgmanite undergoes a high-spin to low-spin transition at *P-T* conditions relevant to the top lower mantle^5^. Of particular interest to our understanding of deep-mantle seismology and geodynamics are the effects of the spin transition on the elasticity (e.g., sound velocities, equation of states (EoS), and seismic anisotropies) of lower-mantle minerals, because a thorough knowledge of their elastic properties is essential for interpreting seismic observations as well as for constraining the chemical composition and mineralogy of the region^14^.

In recent years, there have been a number of experimental and theoretical studies on the elasticity of single-crystal ferropericlase across the spin transition at high pressures using various techniques, including Impulsive Stimulated Light Scattering (ISS), Brillouin Light Scattering (BLS), Inelastic X-ray Scattering (IXS), and Density Function Theory (DFT)^1^,^3^,^4^,^24^,^25^. DFT calculations have shown that ferropericlase exhibits significant softening in *V*_*P*_ as well as in the *C*_*11*_ and *C*_*12*_ elastic constants across the spin crossover at lower-mantle *P-T* conditions, although *V*_*S*_ and *C*_*44*_ are not affected by the transition^25^. On the other hand, experimental results on the elasticity across the spin transition differ drastically^1^,^2^,^3^,^4^. In particular, ISS measurements on (Mg_0.94_Fe_0.06_)O up to 60 GPa showed a remarkable reduction in both *V*_*P*_ and *V*_*S*_ across the spin transition^1^, although the reliability of deriving the *V*_*S*_ from the interfacial wave has been questioned. BLS measurements on (Mg_0.9_Fe_0.1_)O up to 81 GPa showed no substantial reduction of the directly-measured *V*_*S*_ across the spin transition, while the combination of the *V*_*S*_ values from BLS and the EoS parameters from X-ray diffraction have shown a *V*_*P*_ softening up to 17% within the spin transition^2^. High-pressure IXS experiments up to 80 GPa on (Mg_0.83_Fe_0.17_)O, which used acoustic phonon dispersions at very high frequencies to extract the velocities, did not reveal any reduction of either *V*_*P*_ or *V*_*S*_ within the spin transition^3^. Furthermore, some of these previous studies have indicated that the spin transition can markedly enhance the elastic *V*_*S*_ splitting anisotropy of ferropericlase such that the low-spin state becomes much more elastically anisotropic as compared to its high-spin counterpart^3^,^4^. The full elastic constants of single-crystal ferropericlase across the spin transition and in the low-spin state can help provide new insight into the effects of the spin transition on thermodynamic and seismic parameters of the sample, but these essential experimental data remain unclear due to the aforementioned technical difficulties. Since different experimental results point to entirely different scenarios for seismic and geochemical models of the lower mantle, seismically homogeneous vs. heterogeneous lower mantle, it remains unclear if the effect of the spin transition on the elasticity of the lower-mantle ferropericlase should be taken into account in our understanding of the seismic models of the lower mantle. Reliable experimental results can also help elucidate recent theoretical predictions of the elasticity and thermodynamics of ferropericlase at high pressures^25^.

Here we have directly measured *V*_*P*_, *V*_*S*_, and the pressure-volume (*P-V*) relation of a single-crystal ferropericlase (Mg_0.92_,Fe_0.08_)O in order to solve for its full elastic constants (*C*_*ij*_) using combined BLS, ISS, and X-ray diffraction (XRD) measurements in a diamond anvil cell (DAC) up to 96 GPa (see Methods for details). The combined experimental results overcome previous technical difficulties and permit direct and reliable evaluation of the full elastic constants and thermoelastic parameters across the spin transition at lower-mantle pressures. Our results show that ferropericlase with 8 at% iron undergoes a spin transition at 40–60 GPa that is associated with changes in elastic constants: *C*_*11*_ softening by a maximum of 16%, and *C*_*12*_ by 70%, whereas *C*_*44*_ does not show any observable reduction across the transition. Within the spin transition, the aggregate *Ks* and *V*_*P*_ calculated from Voigt-Reuss-Hill average of the elastic constants reduces by 38% and 13%, respectively, and the aggregate *V*_*S*_ is not visibly affected by the spin transition. The *V*_*P*_ anisotropy changes to a maximum value of 11%(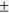
0.7) at 50 GPa, which is midway between the spin transition as compared to 9%(±0.2%) in the *V*_*P*_ anisotropy for the extrapolated high-spin state. The low-spin state also exhibits some elastic behavior distinct from that of the high-spin and mixed-spin states. To decipher the geophysical and geochemical consequences of the spin crossover in the deep mantle, we have modelled elastic and seismic parameters of ferropericlase along an expected lower-mantle geotherm^26^. Our results show that the velocity abnormalities and elastic softening remain significantly strong across the spin crossover in the lower mantle and that the low-spin ferropericlase exhibits significantly enhanced *V*_*P*_ and *V*_*S*_ profiles from that of the extrapolated high-spin state. These results are applied to understand potential seismic and/or chemical heterogeneities induced by the spin transition in the deep lower mantle.

## Results and Discussion

### Experiments and Thermoelastic Modelling

**Figure 1:**
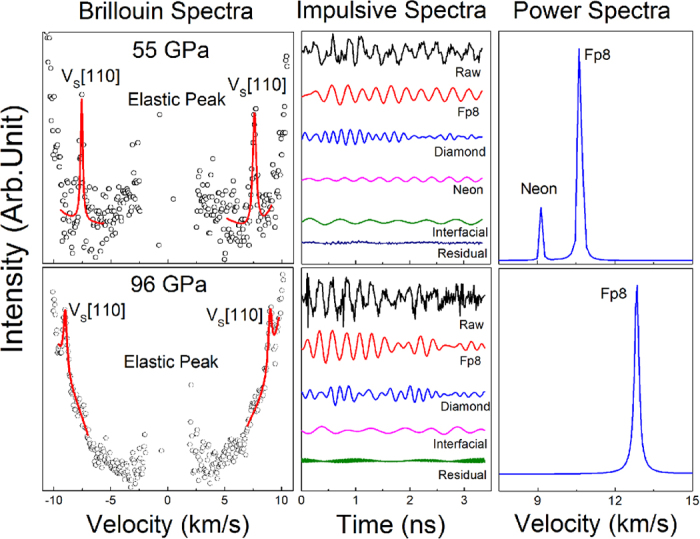
Representative Brillouin light scattering (BLS), impulsive stimulated scattering (ISS), and power spectra of the single-crystal ferropericlase (Mg_0.92_Fe_0.08_)O along [110] crystallographic axis at high pressures. The BLS spectra were used to derive the *V*_*S*_, while the *V*_*P*_ was detected in the ISS spectra. The ISS spectra in the time domain were analyzed and Fourier-transformed to the power spectra in the velocity (frequency) domain to derive the acoustic waves of the sample at high pressures. Neon medium was also observed in the ISS spectra at pressures up to approximately 70 GPa.

**Figure 2:**
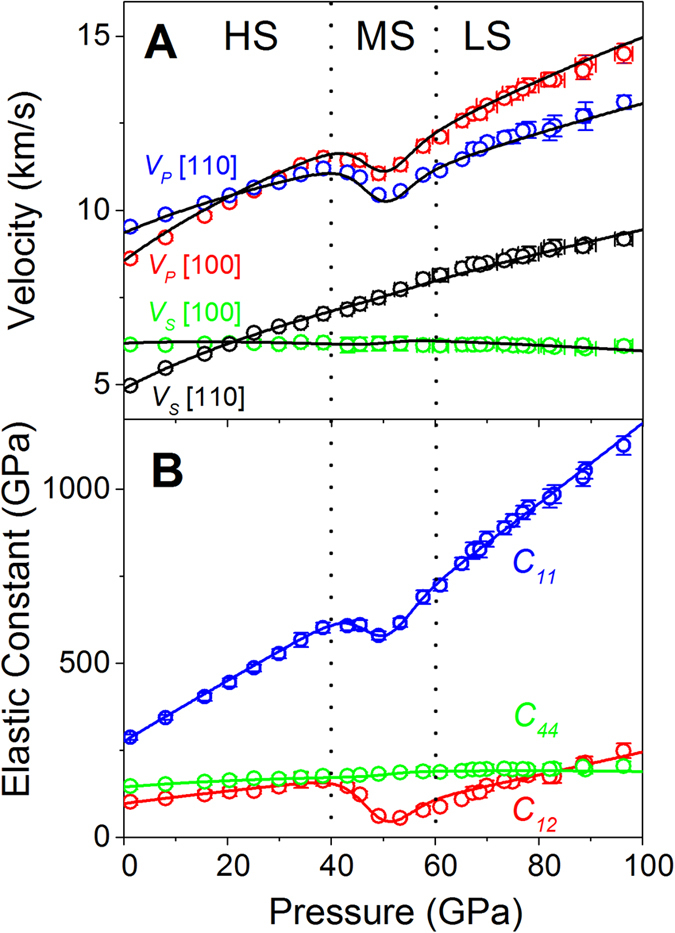
Elasticity of single-crystal ferropericlase (Mg_0.92_Fe_0.08_)O as a function of pressure at 300 K. (**A**) Compressional and shear wave velocities along the [100] and [110] crystallographic axes as a function of pressure. Compressional wave velocities were measured using the ISS technique, while shear wave velocities were measured using the BLS technique. Open circles: experimental data; solid lines: modelled velocity profiles using thermoelastic equations (see SI for details). (**B**) Elastic constants (*C*_*ij*_) as a function of pressure. Open circles: *C*_*ij*_ directly derived from measured compressional and shear wave velocities via Christoffel’s equations; solid lines: modelled *C*_*ij*_ profiles. Vertical dashed lines are plotted to guide the eyes for the high-spin (HS), mixed-spin (MS; HS + LS), and low-spin (LS) regions, respectively (see Supplementary Fig. S2 for details).

**Figure 3:**
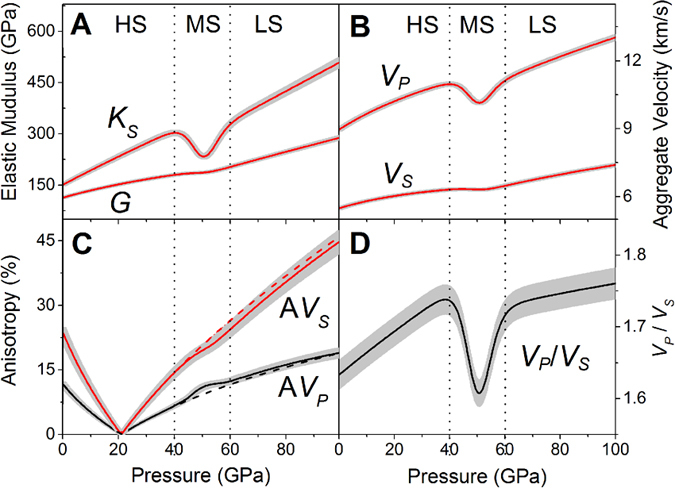
Aggregate bulk and shear moduli *K*_*S*_ and *G*, aggregate velocities, elastic anisotropies and aggregate *V*_*P*_/*V*_*S*_ ratio of ferropericlase (Mg_0.92_Fe_0.08_)O at high pressure and 300 K. (**A**) Adiabatic bulk and shear modulus from Voigt-Reuss-Hill average; (**B**) Aggregate compressional *V*_*P*_ and shear wave velocities *V*_*S*_, where 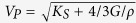 and 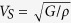; (**C**) Compressional and shear wave anisotropy as a function of pressure; dashed lines are the extrapolated anisotropies for the HS state that are plotted for comparison; (**D**) aggregate *V*_*P*_/*V*_*S*_ ratio. Grey shaded areas represent uncertainties calculated from standard error propagations using the experimentally derived elastic constants. Vertical dashed lines are plotted to guide the eyes for the HS, MS, and LS regions, respectively.

*P-V* relations of single-crystal ferropericlase ((Mg_0.92_, Fe_0.08_)O) in the (100) platelet were measured using synchrotron X-ray diffraction up to 91 GPa at room temperature in a DAC. These results are used to evaluate the EoS parameters, the width of the spin transition, and the fraction of the high-spin (HS) and low-spin (LS) states in ferropericlase^20^,^23^ (Fig. S1 and Fig. S2) (See Methods and SI for details of the experiments and modelling). Analysis of the measured *P-V* curve shows that the spin transition occurs over pressures ranging between 40 GPa and 60 GPa, and is associated with a density increase of 1.2% (0.1%). The derived isothermal bulk modulus at ambient conditions (*K*_*T0*_ ) and its pressure derivative (*K*_*T0*_′) are: *K*_*T0*_ = 152.5 (2.4) and *K*_*T0*_′ = 4.1 (0.2) for the HS state, and *K*_*T0*_ = 161.6 (7.1) with a fixed *K*_*T0*_′ of 4 for the LS state, consistent with previous studies^27^ (Fig. S3). The single-crystal platelet was also used for simultaneously measuring *V*_*S*_ in the BLS experiments and *V*_*P*_ in the ISS experiments along principle [100] and [110] crystallographic axes up to 96 GPa in the Mineral Physics Laboratory of The University of Texas at Austin (Figs 1 and 2); at relatively lower pressures, the *V*_*P*_ and *V*_*S*_ velocities of the platelet were also measured as a function of the azimuthal angles in order to confirm the orientation of the platelets and to further assure the reliability of our measurements as compared with previous studies (Fig. S4). Together with *P-V* results from XRD measurements, the measured *V*_*P*_ and *V*_*S*_ velocities of single-crystal ferropericlase permit direct derivations of the full elastic constants (*C*_*11*_, *C*_*12*_, *C*_*44*_) at high pressures via Christoffel’s equations (Fig. 2). Using the Eulerian finite-strain theory^28^ and a thermoelastic model for the cubic system^25^, we have modelled the elastic constants within the spin transition using formulations reported previously^25^ (See SI for details). Specifically, the elastic compliances *S*_*ij*_ of the crystal across the spin transition are given by:


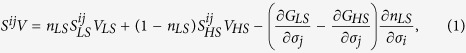


where *V* is the volume, *n*_*LS*_ is the LS fraction, *σ*_*i*_ and *σ*_*j*_ are the *i* th and *j*th stress component, respectively, in the Voigt notation, and *G* is the Gibbs free energy. In this modelling, the low-spin fraction (*n*_*LS*_) and the unit cell volume (*V*) derived from the equation of state, and the elastic constants (*C*_*ij*_) are used to constrain the elastic compliances *S*_*ij*_ according to the relationship between *C*_*ij*_ and *S*_*ij*_ (See SI for details). To further obtain the pressure-dependent EoS parameters for the HS and LS states, respectively, the elastic constants and the aggregate bulk and shear moduli as a function of pressure are derived by fitting the results to the third-order Eulerian finite-strain equation of state (Figs 2 and 3).

Examination of the directly-measured *V*_*P*_ and *V*_*S*_ velocities show that the *V*_*P*_ softens by ~10% maximum in both the [100] and [110] directions within the spin transition, while the *V*_*S*_ along the [100] is slightly enhanced, but the *V*_*S*_ along the [110] is not noticeably affected (Fig. 2A) (see SI for comparison with previous results). Furthermore, the *C*_*11*_ and *C*_*12*_ elastic constants are significantly softened by a maximum of 16% and 70%, respectively, across the spin transition, but *C*_*44*_ is not affected by the spin transition (Fig. 2B). The maximum softening for these parameters occurs at approximately 50 GPa, which is midway within the spin transition where the fraction of the LS state is about 50% (Fig. S2). As reported in previous theoretical calculations^25^, the *C*_*11*_ and *C*_*12*_ softening can be explained by the addition of an energy abnormality as a result of the HS and LS mixing (shown in the last term in Equation (1)). Since *n*(*σ*_4_) is an even function and 
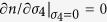
, the last term in Equation (1) vanishes such that the *C*_*44*_ softening is not expected to occur across the spin crossover^25^. Our results thus confirm theoretical predictions of the elasticity across the spin transition in ferropericlase at high pressures. The derived elastic constants are used to calculate *V*_*S*1_, *V*_*S*2_, and *V*_*P*_ velocities as a function of propagation directions (***n***) by solving Christoffel’s equation det|*C*_*ijkl*_*n*_*j*_*n*_*l*_ – *ρV*^2^*δ*_*ik*_| = 0 (Fig. S7). At ambient conditions, the single-crystal ferropericlase exhibits the slowest *V*_*P*_ in the [100] direction and the fastest *V*_*P*_ in the [111] direction, while the *V*_*S*_ is slowest in the [110] direction and fastest in the [100] direction. This anisotropic behavior is reversed at pressures of approximately 21 GPa, above which the *V*_*P*_ minimum and maximum are along the [111] and [100], respectively, while the *V*_*S*_ minimum and maximum are along the [100] and [110] directions. The velocities vary significantly with the propagation direction at pressures above approximately 40 GPa, indicating that single-crystal ferropericlase exhibits strong *V*_*P*_ and *V*_*S*_ anisotropy. The velocity anisotropy factor (*A*) is defined as *A* = (*V*_*max*_ – *V*_*min*_)/2(*V*_*max*_ + *V*_*min*_) × 100%, where *V*_*max*_ and *V*_*min*_ are the maximum and minimum velocities, respectively^29^. For *V*_*P*_, the anisotropy is defined as the difference between the maximum and minimum velocities in corresponding propagation directions, while the *V*_*S*_ splitting anisotropy is defined as the velocity difference between the maximum and minimum velocities for two orthogonally polarized *V*_*S*_ velocities along corresponding propagation directions. Our results show that the *V*_*P*_ anisotropy and the *V*_*S*_ splitting anisotropy are 11.5% and 23.5% at ambient conditions, decreasing to almost zero at approximately 21 GPa, and then increasing monotonically up to 40 GPa. The *V*_*P*_ anisotropy increases to ~11% at 50 GPa, which is midway between the spin transition, while the anisotropy of the extrapolated HS state is approximately ~9%. Furthermore, the *V*_*S*_ splitting anisotropy continuously increases with increasing pressure but slightly deviates from the extrapolated HS state counterpart starting with the spin transition (Fig. 3C and Fig. S8). Within the experimental uncertainties in this study, the effect of the spin transition on the *V*_*S*_ splitting anisotropy can be considered negligible (Fig. 3C). Contrary to previous studies^4^, our results show that the spin transition is actually associated with a slightly enhanced *V*_*P*_ anisotropy (Fig. S8). Furthermore, our results clearly show that the LS ferropericlase exhibits some unique elastic behavior distinct from that of the HS state, including an enhanced pressure derivative of *C*_*11*_, *C*_*12*_, and *C*_*44*_, implying an enhanced pressure dependence of aggregate *K*_*S*_, *V*_*P*_, and *G* (Figs 2 and 3A,B; Table S2). In particular, the spin transition is associated with a significant reduction of the aggregate *V*_*P*_*/V*_*S*_ ratio via the aggregate *V*_*P*_ softening since *V*_*S*_ softening does not visibly occur within the spin transition; this ratio is reduced from 1.75 at approximately 40 GPa at the onset of the transition to 1.6 at approximately 50 GPa midway within the transition (Fig. 3B,D). That is, the LS state exhibits a maximum of approximately 12% reduction in the *V*_*P*_*/V*_*S*_ ratio as compared to that of the extrapolated HS state within the spin transition. Such a reduction in the *V*_*P*_*/V*_*S*_ ratio manifests an abnormal Poisson’s ratio within the spin transition and into the LS state.

### Elasticity of ferropericlase along an expected geotherm: implication for lower-mantle seismic heterogeneities

**Figure 4:**
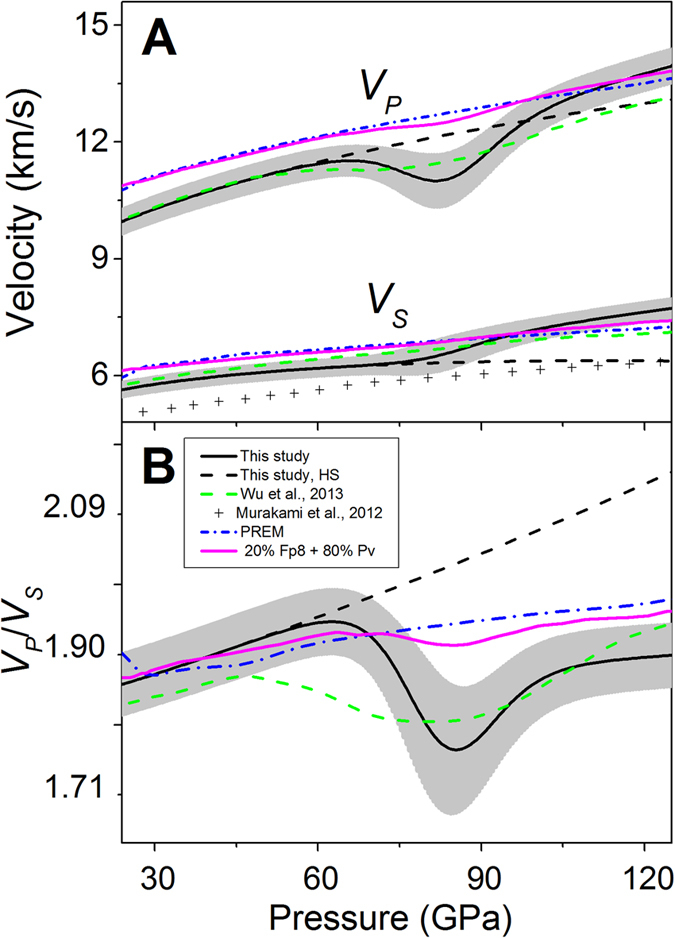
Modelled seismic velocities and *V*_*P*_*/V*_*S*_ ratio of ferropericlase ((Mg_0.92_Fe_0.08_)O) along an expected lower-mantle geotherm. (**A**) Aggregate compressional and shear wave velocities. These results are calculated from the single-crystal elastic constants. (**B**) Calculated *V*_*P*_*/V*_*S*_ ratio. Solid lines: modelled seismic parameters with uncertainties shown as grey areas; black dashed lines: modelled parameters for the high-spin state; green dotted lines: theoretical *V*_*P*_ and *V*_*S*_ values of ferropericlase ((Mg_0.875_Fe_0.125_)O)^25^; crosses: experimental results with 17% iron^14^. PREM seismic parameters are plotted as blue dotted dashed lines for comparison^6^; The magenta lines are the modelled velocity profiles assuming that the lower mantle is composed of 20% ferropericlase (fp8) and 80% bridgmanite (Pv)^14^.

To understand the effects of the spin transition on the elasticity of ferropericlase at relevant *P-T* conditions of the lower mantle^22^,^30^, we have modelled the elastic constants of ferropericlase along an expected lower-mantle geotherm up to approximately 125 GPa using thermoelastic models and a previously reported spin crossover diagram^23^,^25^. The geotherm profile at the core-mantle boundary conditions has not been considered in our modelling here. The thermal EoS parameters of ferropericlase with 25 at% iron in a previous experimental report are linearly scaled back for the compositional effects of FeO in MgO to construct the spin crossover diagram for our ferropericlase with 8 at% iron at high *P-T* (See SI for details). Our modeled results show that the spin crossover of ferropericlase with 8 at% iron occurs between 65 and 105 GPa along the geotherm. The temperature derivatives of the elastic constants for pure MgO^31^ are combined with our high-pressure elasticity results (Table S1 and Table S2) to account for the high *P-T* effects of FeO solid solution for the HS and LS states (Fig. S9). These modelled results show that the effects of the spin crossover on the elastic and seismic parameters along an expected adiabatic geotherm remain profound, even though the spin crossover is broadened by high temperatures (Fig. 4 and Fig. S9). In particular, *C*_*11*_ and *C*_*12*_ exhibit 15% and 60% maximum reduction, respectively, within the spin crossover at approximately 85 GPa that corresponds to 1900 km in depth. The *V*_*P*_ anisotropy increases to 18.6% at ~85 GPa within the spin transition (a 23% increase in the anisotropy as compared to the extrapolated HS state reference), while the pressure-dependent *V*_*S*_ anisotropy is lower than that of the HS counterpart in the LS state (Fig. S9 B). Compared to the HS state reference, the aggregate *V*_*P*_ decreases by 10% while the *V*_*P*_/*V*_*S*_ ratio drops by 13% within the spin crossover (Fig. 4). On the other hand, the aggregate *V*_*P*_ and *V*_*S*_ profiles of the LS ferropericlase are significantly higher than that of their HS state counterparts (Fig. 4A). Using the HS state as the reference, we have calculated the deviations of a number of seismic parameters across the spin crossover along an expected mantle geotherm. These results show that the spin crossover produces *V*_*P*_ and *V*_*S*_ velocities and anisotropies, *V*_*P*_/*V*_*S*_ ratio, and Poisson’s ratio that vary as a function of the low-spin fraction as compared to the extrapolated HS state. Specifically, the *V*_*P*_, *V*_*P*_/*V*_*S*_ ratio, and Poisson’s ratio are significantly reduced within the spin crossover, whereas the LS state exhibits enhanced *V*_*P*_ and *V*_*S*_ velocities as well as reduced *V*_*P*_/*V*_*S*_ and Poisson’s ratio (Fig. S10).

Our modelled velocity profiles show that the *V*_*P*_ profile of ferropericlase with 8 at% iron is significantly reduced by a maximum of 10% within the spin crossover at approximately 1900 km in depth along an expected mantle geotherm, while the *V*_*S*_ profile is slightly enhanced with increasing fraction of the LS state as compared to the extrapolated HS state (Fig. 4). That is, the *V*_*P*_/*V*_*S*_ ratio is also significantly reduced with the spin crossover. On the other hand, the *V*_*P*_ and *V*_*S*_ profiles of the LS ferropericlase are higher than those of the extrapolated HS state as well as the PREM model toward the lower parts of the lower mantle. Considering that ferropericlase in the lower mantle may contain approximately 20% FeO and account for approximately 20 vol.% of the lower-mantle in a pyrolite model^9^, our calculated *V*_*P*_ and *V*_*S*_ profiles with 20 vol.% of ferropericlase and 80 vol.% of bridgmanite are fairly consistent with the PREM model at depths from uppermost to mid lower-mantle. On the other hand, the spin crossover in ferropericlase is expected to contribute a few percent reduction in *V*_*P*_ and *V*_*P*_/*V*_*S*_ ratio in the middle parts of the lower mantle compared to the PREM seismic model, while the occurrence of the LS ferropericlase would result in enhanced *V*_*P*_ and *V*_*S*_ profiles compared to the seismic model (Fig. 4). Since the *V*_*P*_ and *V*_*S*_ velocities of ferropericlase behave quite distinctly across the spin transition, our results here also indicate *V*_*P*_/*V*_*S*_ ratio can be used as a more sensitive seismic indicator for probing the spin transition-induced heterogeneities in the lower mantle (Figs 3 and 4).

Based on *V*_*S*_ profiles of polycrystalline ferropericlase and bridgmanite at high *P-T* conditions, it has been proposed that the lower mantle is predominantly made of bridgmanite by 93% in volume, called the perovskitic lower mantle, and that ferropericlase may only account for 7% of the lower-mantle mineralogy^14^. In this scenario, the contributions of the elastic and seismic anomalies of ferropericlase across the spin crossover would play a much smaller role on the overall seismic profiles of the lower mantle such that the associated effects may become seismically insignificant. In our modelling, we have considered the lower-mantle *P-T* conditions along an expected adiabatic geotherm, but the possibility of having a super-adiabatic lower mantle with a steeper thermal gradient than the adiabatic geotherm model is possible^14^. The relatively higher geotherm would widen the spin crossover leading to smaller velocity anomalies within the spin crossover and slower *V*_*P*_ and *V*_*S*_ profiles of the LS ferropericlase in the lower mantle. It also remains to be seen as to how the changes in the partitioning coefficient of iron between bridgmanite and ferropericlase (*K*_D_^Pv-Fp^ = (*X*_Fe_^Pv^/ *X*_Mg_^Pv^)/(*X*_Fe_^Fp^/ *X*_Mg_^Fp^)) across the spin transition can influence the abnormal elasticity in ferropericlase reported here. Previous studies have shown that the *K*_D_^Pv-Fp^ decreases from approximately 0.85 at ~750 km depth to 0.2 at ~1800 km depth in the pyrolitic composition, indicating that Fe^2+^ preferentially partitions into ferropericlase in the middle to lower part of the lower-mantle conditions^32^,^33^. That is, the iron content of lower mantle minerals can be largely influenced by the change of the iron partitioning associated with the occurrence of the spin transition. In this case, the spin crossover of ferropericlase can even occur over a wider range of *P-T* conditions with variable amounts of iron involved, thus spreading seismic and chemical anomalies in ferropericlase that may become too broad to be seismically detectable with our current seismic resolution. Thus far, seismic studies have yet revealed reliable features of the lower mantle that can be associated with the spin transition effects in the region. Future seismic studies of the lower mantle taking the effects of the spin transition as well as temperature and composition parameters into account are needed to decipher the geophysical consequences of the spin transition of ferropericlase in the region. Our results point to potential consequences of the spin transition for seismic heterogeneities in the lower mantle and also highlight a much more complex picture of the elasticity of ferropericlase in the lower mantle, affecting our understanding of seismology, geochemistry, and geodynamics in the region.

## Methods

Single-crystal ferropericlase ((Mg_0.92_,Fe_0.08_)O) was synthesized via inter-diffusion of Fe and Mg between a single-crystal periclase and pre-synthesized (Mg,Fe)O powder in a H_2_/CO_2_ gas-mixing furnace at the Institute for Study of the Earth’s Interior (ISEI) of Okayama University at Misasa. The MgO crystal with a pre-oriented (100) crystallographic plane purchased from the MTI Corporation was cut down to 7 mm in length by 7 mm wide and 0.25 mm thick, and was sandwiched between two layers of compacted polycrystalline (Mg_0.75_Fe_0.25_)O powder approximately 1 mm thick each. The starting sample assemblage was then placed in a Pt holder into the furnace operating at 1350 °C and 10^−2^ Pa oxygen fugacity for approximately 2 weeks. The synthesized single-crystal ferropericlase was then extracted and polished for further sample analyses. Electron microprobe and X-ray diffraction analyses of the sample at The University of Texas at Austin showed that the sample was chemically homogeneous with the chemical composition of (Mg_0.92_Fe_0.08_)O and a unit cell parameter of *a* = 4.1996 (4) Å. The (100)-oriented sample was double polished down to approximately 15 μm thick, and cut into squared platelets 50–80 μm in length for high-pressure DAC experiments. The orientation of the platelet was confirmed by the single-crystal X-ray diffraction patterns at ambient and high pressure (Fig. S1B).

High-pressure X-ray diffraction patterns were collected from the single-crystal sample at room temperature in a DAC at the Sector 13-BMD of the GSECARS of the Advanced Photon Source (APS), Argonne National Lab (ANL) (Fig. S1). A pair of diamond anvils with 200 μm culets were used to pre-indent a rhenium gasket with an initial thickness of 250 μm to approximately 25 GPa (or approximately 25 μm thick). Consequently, a hole of 120 μm was drilled in the pre-indented area and used as the sample chamber. A piece of the platelet 50 μm in length was loaded, together with Au powder as the pressure calibrant and Ne as the pressure medium, in a short symmetric DAC. An incident X-ray beam with a wavelength of 0.3344 Å and focused size of 20 μm (FWHM) in diameter was used for the diffraction experiments (Figs S1 and S2). X-ray diffraction patterns of the sample were collected at pressure intervals of 1–3 GPa up to 91 GPa by a MAR CCD by continuously rotating the DAC around the vertical axis of the sample stage by 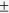
15° (Fig. S2). The unit cell parameters and their uncertainties for the sample were calculated based on four sets of the diffraction peaks corresponding to {200}, {220}, {400}, and {420} equivalent reflections (Fig. S1). The uncertainties of the unit cell parameters are typically in the order of 0.04% and are approximately 0.08% at the highest pressure of 91 GPa, indicating that the sample remained sufficiently high quality for the X-ray diffraction, BLS, and ISS experiments. Analysis of the XRD patterns of the sample also confirmed that the crystal was indeed oriented in the (100) crystallographic plane within approximately 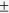
1° angular uncertainty.

High-pressure BLS and ISS experiments were performed on the single-crystal ferropericlase at up to 96 GPa in a short symmetric DAC in the Mineral Physics Laboratory of The University of Texas at Austin. Ultralow birefringence and microscopically defect-free diamond anvils were selected for these experiments using a petrographic microscope under crossed-polar, because we had observed that the pulsed laser of the ISS system with a 1064 nm wavelength could potentially damage diamond anvils having defects and high strained areas. Similar to the sample preparation in the XRD experiments, a pair of diamond anvils with 200 μm culets was used to pre-indent a rhenium gasket and a hole of 120 μm was drilled and used as the sample chamber. A piece of the platelet 50–70 μm in length was loaded, together with a few ruby spheres as the pressure calibrant and Ne as the pressure medium, in a short symmetric DAC. Two runs were conducted for the BLS and ISS experiments (Table S1). Pressure uncertainties were determined from measured ruby fluorescence spectra before and after the BLS and ISS measurements. BLS spectra of the sample were collected from the (100) platelet along the [100] and [110] crystallographic directions in the transmitted geometry with a pressure interval of 3–5 GPa up to 96 GPa (Figs 1 and 2). The BLS system is equipped with a Coherent Verdi V2 laser operating at 532 nm wavelength and 600 mW laser power, together with a JRS interferometer and an APD detector (Count-10B Photo Counting Module with approximately 5 cps from Laser Components, Inc.). The focused laser beamsize at the sample position was approximately 20 μm in diameter while the scattering angle of the BLS system was set at 48° and calibrated against SiO_2_ glass and purified water standards. The data collection time was typically 1 hour at pressures below 50 GPa and 2 hours at higher pressures. Analyses of the Brillouin spectra using OriginPro 9.1 software showed strong *V*_*P*_ and *V*_*S*_ peaks with high signal-to-noise ratios at pressures below 20 GPa, while only the *V*_*S*_ peak of the sample was observed at higher pressures as the *V*_*S*_ peak of the diamond anvils saturated the *V*_*P*_ peak of the sample. The measured *V*_*P*_ and *V*_*S*_ velocities of the sample at pressures below 20 GPa were used to derive the full elastic constants of the crystal and also to cross check the reliability of the ISS results at lower pressures (see further discussion below).

High-pressure ISS spectra were also collected from the single-crystal sample along the [100] and [110] crystallographic directions up to 96 GPa (Fig. 1). The ISS system is a pump-and-probe technique that is equipped with the pump laser with a 1064 nm wavelength and a pulse width of 15 ps and the probe laser with a 532 nm wavelength. The pump laser from Talisker of the Coherent Company was split into two beams which were then recombined at the sample position with a crossing angle of 20.3° and a beam size of 30 μm. The probe laser was delayed by an Aerotech linear stage as long as 20 ns, while the diffracted ISS signals were collected by a photodiode detector. The data collection time for each ISS spectrum was typically 2 hours. Using MATLAB and OriginPro 9.1 softwares, we implemented the Burg method to analyze the time-domain ISS spectra in order to derive the frequency-domain power spectra and the acoustic wave velocities of the sample at high pressures (Fig. 1). The derived *V*_*P*_ values from ISS measurements are consistent with those from the BLS measurements at pressures below 20 GPa, confirming the calibration and reliability of both techniques. The interfacial waves were also observed in most of the ISS experiments, but their signals were much weaker than that of the longitudinal acoustic waves; in fact, the interfacial waves were too weak to be observed in our analyses in some experiments. Given the uncertainties involved in interpretation of the interfacial wave results from ISS experiments, we have only used the *V*_*P*_ from the ISS measurements and the *V*_*S*_ from the BLS experiments, together with the density results from XRD measurements, to derive full elastic constants of the single-crystal ferropericlase at high pressures.

## Additional Information

**How to cite this article**: Yang, J. *et al.* Elasticity of Ferropericlase across the Spin Crossover in the Earth's Lower Mantle. *Sci. Rep.* **5**, 17188; doi: 10.1038/srep17188 (2015).

## Supplementary Material

Supplementary Information
