# Supplementary material for: Elasticity of Ferropericlase across the Spin Crossover in the Earth’s Lower Mantle
Source: Sci Rep. 2015 Dec 1;5:17188. doi: 10.1038/srep17188 (PMC4664863; doi:10.1038/srep17188)
Supplement: Supplementary Information [file srep17188-s1.pdf]

Supplementary Material

Elasticity of Ferropericlase across the Spin Crossover in the Earth's Lower Mantle

Jing Yang[a], Xinyue Tong[a], Jung-Fu Lin[a,b,1], Takuo Okuchi[c], Naotaka Tomioka[c]

[a]Department of Geological Sciences, Jackson School of Geosciences, The University of Texas at Austin, Austin, TX 78712, USA

[b]Center for High Pressure Science and Technology Advanced Research (HPSTAR)

[c]Institute for Study of the Earth's Interior, 827 Yamada, Misasa, Tottori, 682-0193 Japan

Supplementary Text

Modelling the Fraction of the High-Spin and Low-Spin States in Ferropericlase

Following the modelling procedures reported in previous studies[1-3], we have used the pressure-volume[4] relations from our X-ray diffraction measurements to evaluate the fraction of the high-spin (HS) and low-spin (LS) states of iron in ferropericlase $(Mg_{0.92}Fe_{0.08})O$ as a function of pressure and temperature (*P*-*T*) (Figs. S1 and S2). The *P*-*V* data of the ferropericlase was initially compared with the *P*-*V* relation of the end-member MgO as a starting reference at high pressures and room temperature[4]. The comparison permits us to clearly evaluate the volume reduction over the pressure range across the spin transition[2]. Since the HS ferropericlase exhibits a similar equation of state (EoS) behavior to that of MgO, such comparison also helps establish the EoS parameters for the HS state[2]. With the width of the transition and the thermal EoS parameters of the HS state initially established, the fraction of the LS state (n_{LS}) at a given *P*-*T* condition can be obtained using the following equations[1,3] (Fig. S2):

$$n_{LS} = \frac{1}{1 + \exp\left(\frac{\Delta G(P,T)^*}{T}\right)} \qquad [1]$$

$$\Delta G(P,T)^* = b_0(T) + b_1(T)P_n \qquad [2]$$

$$P_n = \frac{P - P_{HS}}{P_{LS} - P_{HS}} \qquad [3]$$

where $\Delta G(P,T)^*$ is the difference in Gibbs free energy between the LS and HS states, P_n is the normalized pressure as determined by the ending pressure of the HS state (P_{HS}) and the onset pressure of the LS state (P_{LS}), and b_0 and b_1 are two temperature-dependent constants. Using the non-linear least squares fit of the n_{LS} to the *P*-*V* data at 300 K, we have obtained b_0 = 1220 (25) and b_1 = −2341 (46).

Based on the solid-solution mixing of the HS and LS states in the ferropericlase lattice as well as the derived LS fraction (n_{LS}), the unit cell volume of ferropericlase (V) across the spin transition is expressed as the ratio between the unit cell volume of the HS state (V_{HS}) and the LS state (V_{LS}) at a given pressure and 300 K (Fig. S2)[3]:

$$V = (1 - n_{LS})V_{HS} + nV_{LS} \quad [4]$$

It follows that the isothermal bulk modulus (K_T) of the system across the spin transition can be described using the ratio of the HS and LS states[3]:

$$\frac{V}{K_T} = n_{LS}\frac{V_{LS}}{K_{LS}} + (1 - n_{LS})\frac{V_{HS}}{K_{HS}} - (V_{LS} - V_{HS})\left(\frac{\partial n_{LS}}{\partial P}\right)_T \quad [5]$$

$$V_{\emptyset} = \sqrt{\frac{K_T}{\rho}} \quad [6]$$

where K_{HS} and K_{LS} are the K_T of the HS and LS state, respectively, ρ is the density, and V_{Φ} is the bulk sound velocity (Figs. S2 and S3).

Derivation of the Full Elastic Constants of the Single-Crystal Ferropericlase

The ferropericlase platelet with (100) orientation allows us to measure V_P velocities as well as V_S velocities with <110> polarization along principle [100] and [110] crystallographic axes using the ISS and BLS techniques, respectively, in a DAC. Together with the P-V data from synchrotron X-ray diffraction measurements (Figs. S1 and S2), here we have combined the V_S data from BLS measurements and the V_P data from ISS measurements (Fig. 1) to derive the elastic constants (C_{11}, C_{12}, C_{44}) of the single-crystal ferropericlase using the following equations[5] (Fig. 2 and Fig. S6):

$$V_P\ [100] = (C_{11}/\rho)^{1/2} \quad [7]$$

$$V_S\ [100] < 110 >= (C_{44}/\rho)^{1/2} \quad [8]$$

$$V_P\ [110] = [(C_{11} + C_{12} + 2C_{44})/2\rho]^{1/2} \quad [9]$$

$$V_S\ [110] < 110 >= [(C_{11} - C_{12})/2\rho]^{1/2} \quad [10]$$

where [*uvw*] represents the crystallographic direction for the acoustic wave propagation, and <*uvw*> indicates the polarization direction. Since the method for deriving the full elastic constants involves multiple experimental data sets and the use of multiple equations listed above, the elastic constants reported here are derived from internally-consistent numerical iterations through minimization of the uncertainties in the derived parameters using the aforementioned equations as well as the finite-strain equations discussed below[6]. In the finite-strain modelling, the pressure derivatives of K_S and G at a given temperature ($(\partial K_S/\partial P)_T$ and $(\partial G/\partial P)_T$) are obtained by fitting the moduli at high pressure using the third-order Eulerian finite-strain equation of state (EoS)[7]:

$$K_S = K_{S0}(1+2f)^{5/2}\{1 + [3(\partial K_S/\partial P)_T - 5]f\} \quad [11]$$

$$G = (1+2f)^{5/2}\{G_0 + [3(\partial G/\partial P)_T K_{S0} - 5G_0]f\} \quad [12]$$

$$f = \left(\frac{1}{2}\right)[(V_0/V)^{\frac{2}{3}} - 1] \quad [13]$$

where f is the Eulerian strain, and V_0 and V are the unit-cell volumes at ambient conditions and high pressures, respectively. The derived K_S and $(\partial K_S/\partial P)_T$ are converted to the isotherm bulk modulus (K_T) and its pressure derivative at constant temperature ($(\partial K_T/\partial P)_T$) using the following thermodynamic relations[8]:

$$K_{T0} = K_{S0}/(1+\alpha\gamma T) \quad [14]$$

$$(\partial K_T/\,\partial P)_T = (1+\alpha\gamma T)^{-1}[\left(\frac{\partial K_S}{\partial P}\right)_T - (\frac{\gamma T}{K_{T0}})(\partial K_T/\partial T)_P] \quad [15]$$

where $(\partial K_T/\partial T)_P$ is the temperature derivative of the K_T at constant pressure, K_{T0} is the isothermal bulk modulus at ambient conditions, α is the thermal expansion coefficient, and γ is the Grüneisen parameter. Literature values for these parameters ($(\partial K_T/\partial T)_P = -0.017$ GPa K^{-1}, $\alpha = 3.76\times10^{-5}$ K^{-1}, and $\gamma = 1.443$) are used for the conversion[2,4]. The aforementioned procedures were iterated numerically until both K_T and $(\partial K_T/\partial P)_T$ values were self-consistent with the *P-V* data. The derived K_T and $(\partial K_T/\partial P)_T$ values were then used to construct the isothermal EoS of the ferropericlase at high *P-T*.

We have also followed procedure reported in the literature to model the C_{ij} of ferropericlase across the spin transition as a function of pressure[9]. Initially, the Eulerian finite-strain theory is applied to model the C_{ij} of the HS state up to 40 GPa and the LS state above 60 GPa, respectively, at ambient temperature. These elastic constants and their pressure derivatives for the HS and LS states are then used to evaluate the C_{ij} of the mixed-spin (MS) state using the derived n_{LS} and thermoelastic equations described below in which the elastic compliances, S_{ij}, at a given *P-T* condition are defined as[9]:

$$S^{ij} = -\frac{1}{V}\frac{\partial^2 G}{\partial\sigma_i\partial\sigma_j}|_{P,T} \quad [16]$$

where G is the total Gibbs free energy of the system, and σ_i and σ_j are the i_{th} and j_{th} stress components, respectively, in the Voigt notation. The elastic compliances for the cubic ferropericlase are given as[9]:

$$S^{11}V = nS^{11}_{LS}V_{LS} + (1-n)S^{11}_{HS}V_{HS} - 1/9(V_{LS} - V_{HS})\frac{\partial n}{\partial P} \quad [17]$$

$$S^{12}V = nS^{12}_{LS}V_{LS} + (1-n)S^{12}_{HS}V_{HS} - 1/9(V_{LS} - V_{HS})\frac{\partial n}{\partial P} \quad [18]$$

$$S^{44}V = nS^{44}_{LS}V_{LS} + (1-n)S^{44}_{HS}V_{HS} \quad [19]$$

The relationships between the elastic constants and the compliances are described as[10]:

$$C_{11} = \frac{S^{11} + S^{12}}{(S^{11} - S^{12})(S^{11} + 2S^{12})} \quad [20]$$

$$C_{12} = \frac{-S^{12}}{(S^{11} - S^{12})(S^{11} + 2S^{12})} \quad [21]$$

$$C_{44} = \frac{1}{S^{44}} \quad [22]$$

Comparison of the modelled elastic constants with experimental results shows a good agreement with each other in the HS, MS, and LS states, validating the thermoelastic theory for the elasticity of ferropericlase across the spin transition[9] (Fig. 2).

Comparison of the Velocity Results at High Pressures

Here we compare our results with the ones reported in previous studies using similar BLS and ISS techniques. Our measured V_S values along [100] and [110] directions are mostly consistent with those in previous BLS measurements for ferropericlase with 10 at.% iron for the high-spin state [11], but are slightly higher within the spin transition and in the low-spin state (Fig. S5). The difference within the spin transition can be explained as a result of different iron contents in these measurements as higher iron content is expected to contribute to a stronger effect on the velocity. Comparison of our measured V_P with previous BLS measurements below 20 GPa shows great consistency within uncertainties[12], whereas there is a significant discrepancy within the spin transition and in the low-spin state (Fig. S5). Our present results show a stronger V_P softening within the spin transition and a lower V_P in the low spin state than that in the previous ISS measurements for ferropericlase with 6% iron. These differences may be explained by the different iron contents, pressure media, as well as experimental uncertainties including the orientation of the crystal used in the experiments.

Modelling Thermoelastic Parameters across the Spin Crossover in the Lower Mantle

Using the experimentally-derived thermal EoS parameters and the elastic constants at high pressures and 300 K, we have further modelled the elasticity of ferropericlase ($(Mg_{0.92}Fe_{0.08})O$) at high *P*-*T* conditions relevant to the lower mantle along an expected geotherm[13] (Fig. 4 and Fig. S9). Literature results for the spin crossover of ferropericlase ($(Mg_{0.75}Fe_{0.25})O$) are used to construct the spin crossover diagram for our ferropericlase with 8 at.% iron. To reconstruct the spin crossover diagram for our sample, the spin transition pressure of 40-60 GPa at 300 K is used for our ferropericlase sample, instead of 50-75 GPa for ferropericlase with 25 at.% Fe content[2]. Based on previous studies[1,14], elevated temperature widens the spin crossover toward higher pressures. Assuming the thermoelastic properties of ferropericlase can be scaled linearly as a function of the iron concentration, the parameters b_0 and b_1 in the equation (2) can be derived from the non-linear least squares fit to the derived n_{LS} as a function of *P*-*T*:

$$b_0 = -262.5 + 4.9T - 3.0 \times 10^{-4}T^2 \quad [23]$$

$$b_1 = 3155.1 - 16.4T - 8.5 \times 10^{-4}T^2 \quad [24]$$

Together with the literature values for the thermal expansion coefficient of ferropericlase in the HS and LS state (α_{HS} and α_{LS})[2] and the temperature derivative of the elastic constants[15], we have used the modelled spin crossover diagram and the elastic compliances at high pressures and 300 K to calculate the elastic constants at high *P*-*T* using MATLAB (Fig. 4 and Fig. S9)[9].

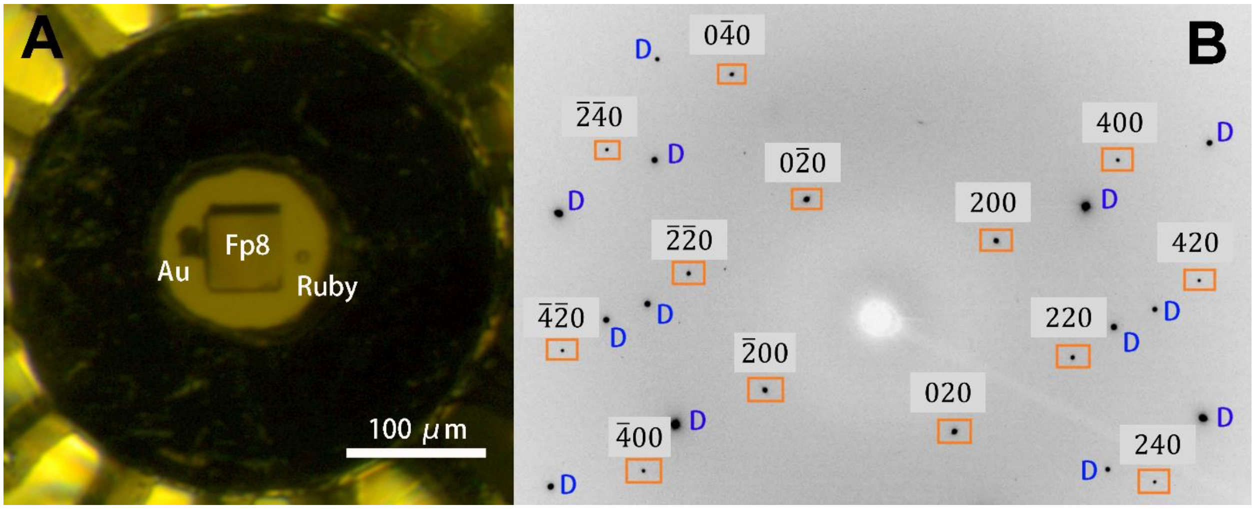

Fig. S1. Representative sample image and X-ray diffraction pattern of the single-crystal ferropericlase $(Mg_{0.92}Fe_{0.08})O$ at high pressure. (A) Ferropericlase (100) platelet, together with Au and a ruby sphere calibrant[16], loaded into a diamond anvil cell having Ne pressure medium at 13 GPa; (B) Representative X-ray diffraction pattern of the single-crystal ferropericlase at 13 GPa. The diffraction pattern was taken by rotating the diamond cell ±15° about the vertical axis of the sample stage. D: diffraction spots from the diamond anvil.

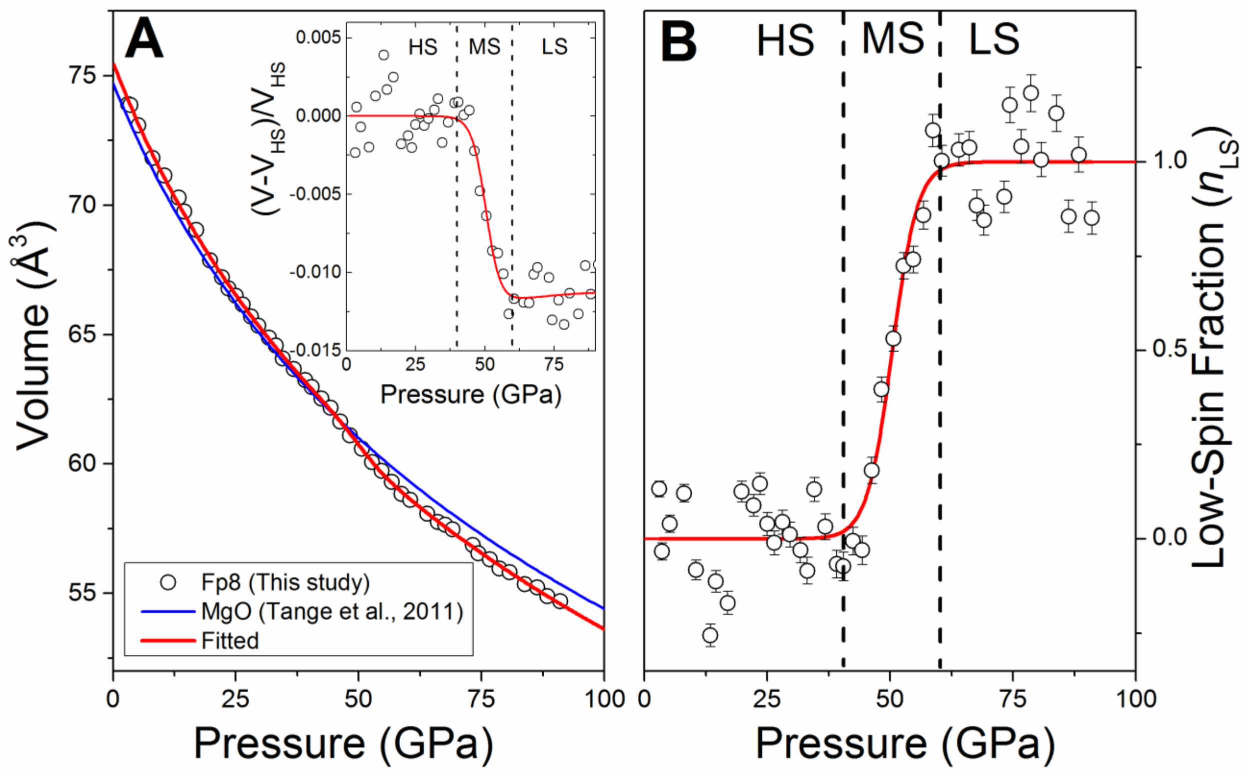

Fig. S2. Pressure-volume relation and the derived low-spin fraction of iron in the single-crystal ferropericlase $(Mg_{0.92}Fe_{0.08})O$ at high pressures. (A) Pressure-volume relation of ferropericlase. Open circles: unit cell volumes as a function of pressure from experimental X-ray diffraction measurements at 300 K; red line: Birch-Murnaghan EoS fit to the experimental data; blue line: pressure-volume curve of the end-member MgO plotted for comparison[4]. The inserted figure shows the volume reduction across the spin transition using the EoS of the high-spin ferropericlase as the reference[2]; (B) Modelled low-spin fraction of the ferropericlase as a function of pressure. The experimental data (open circles) are modelled to derive the low-spin fraction (red line) based on the procedures reported previously[1,2,17]. Vertical dashed lines are plotted to guide the eyes for the high-spin (HS), mixed-spin (MS), and low-spin (LS) regions, respectively.

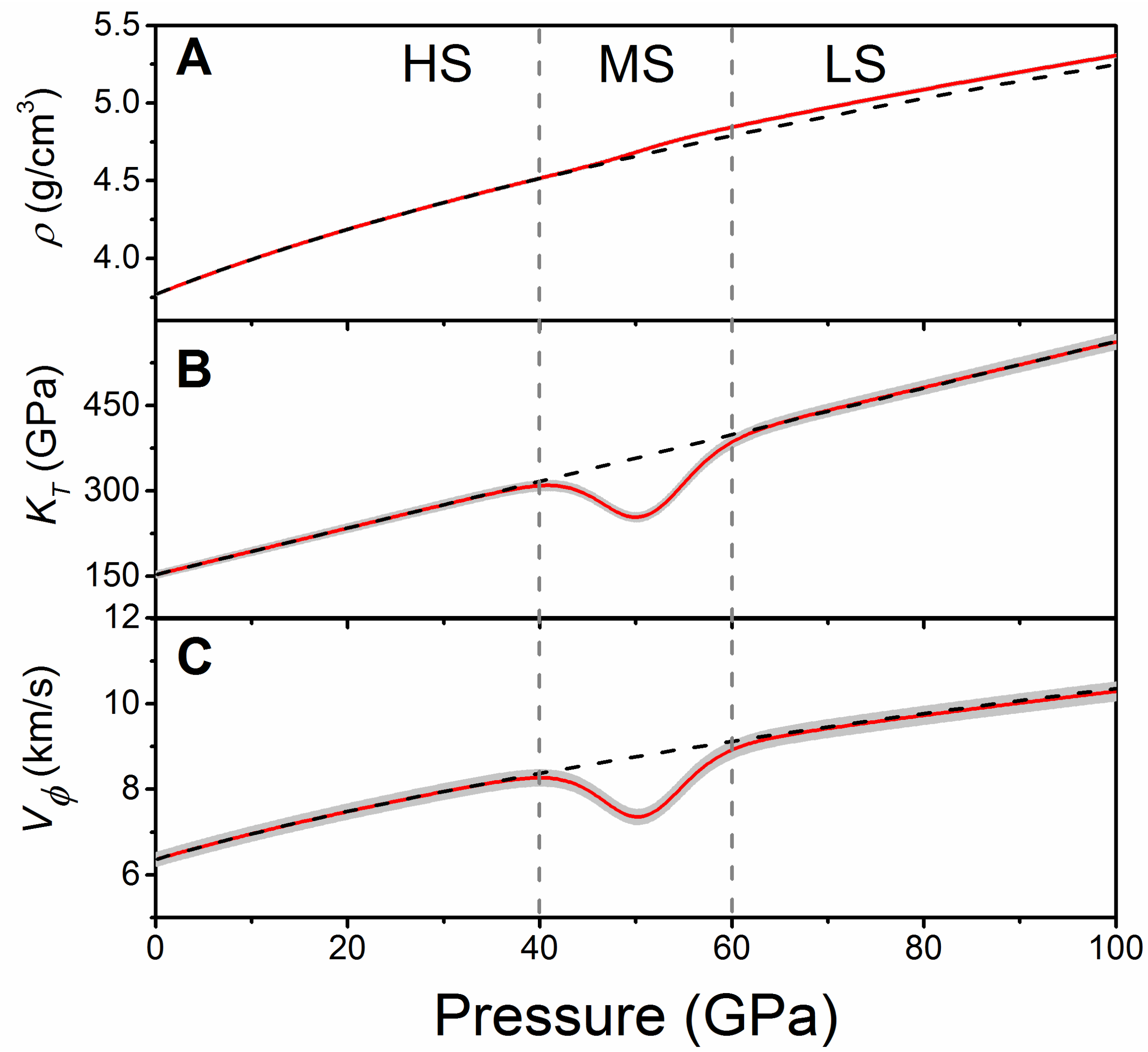

Fig. S3. Equation of state parameters of the single-crystal ferropericlase $(Mg_{0.92}Fe_{0.08})O$ at high pressures and 300 K. (A) Density (ρ); (B) Isotherm bulk modulus (K_T); (C) Bulk sound velocity (V_Φ). Red solid lines: modelled EoS parameters across the spin transition; dash lines: extrapolated EoS parameters for the HS ferropericlase. Grey shaded areas show the uncertainties of the parameters calculated from standard error propagations. Error bars (grey shaded areas) for the density are too small to be shown in the figure.

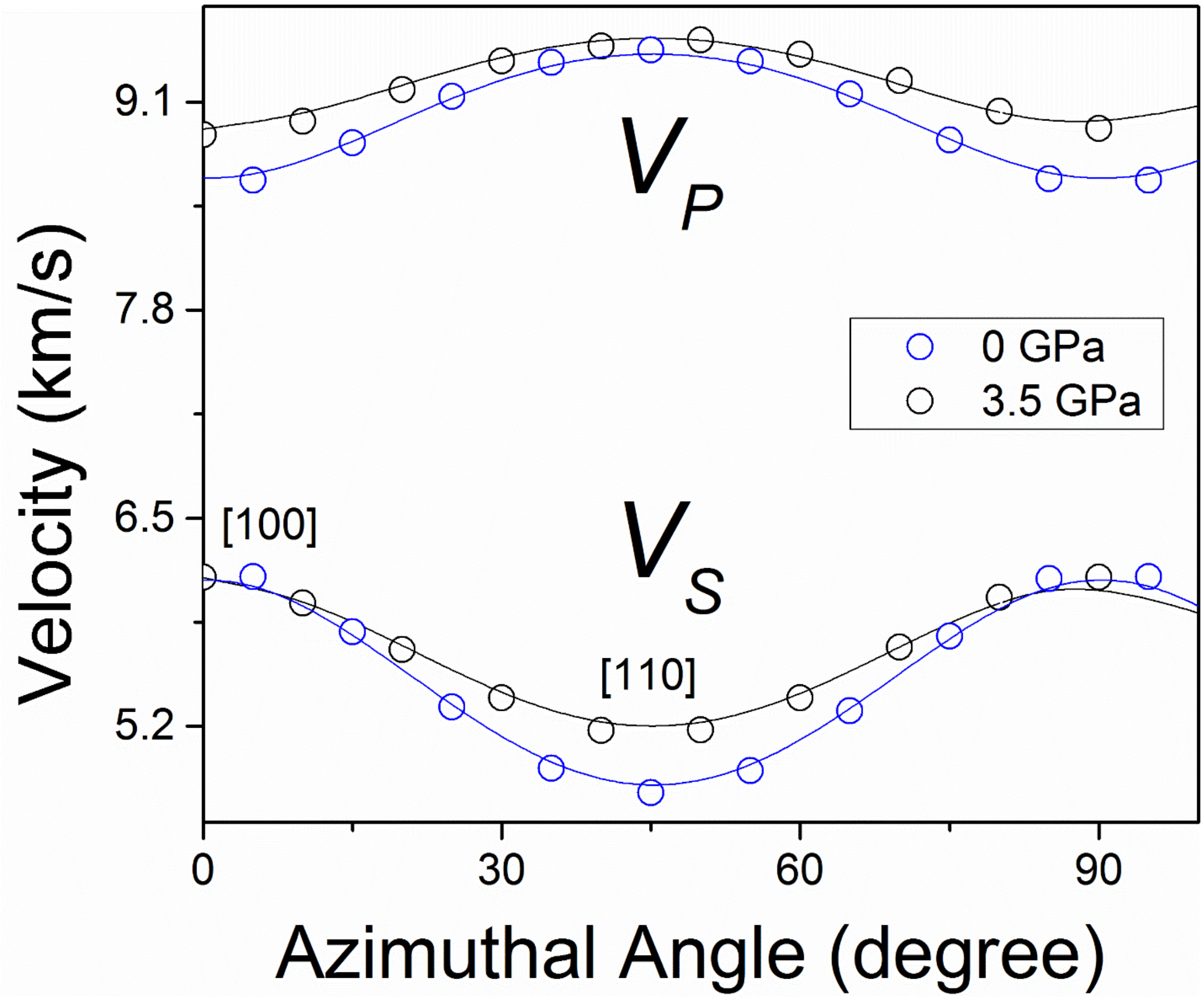

Fig. S4. Compressional (V_P) and shear (V_S) wave velocities of ferropericlase in the (100) platelet as a function of azimuthal angle at ambient conditions (blue circles) and 3.5 GPa (black circles). Uncertainties of the velocities are smaller than the size of the symbols and are not shown for clarity. Solid lines represent modelled velocity profiles from the best fit to the single-crystal elastic constants for each given pressure. These orientation-dependent measurements also help use to locate the [100] and [110] directions for the (100) platelet.

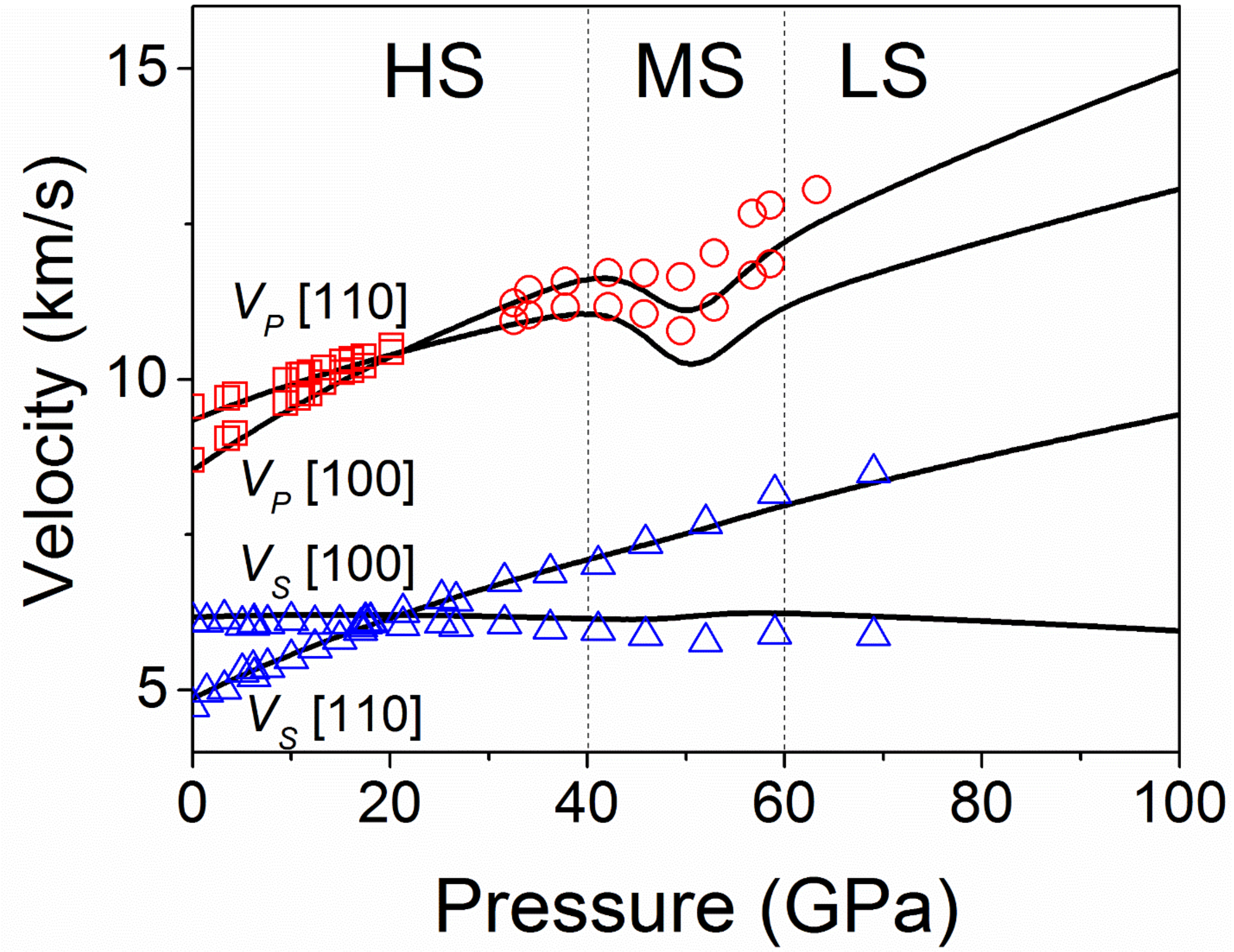

Fig. S5. Comparison of our results with previously reported velocities of ferropericlase at high pressures. Solid lines are modelled experimental results in this study; red squares are BLS measurement for X_{Fe} = 0.06 up to 20 GPa[12]; red circles are ISS measurement for X_{Fe} = 0.06 up to 60 GPa[18]; blue triangles are BLS measurement for X_{Fe} = 0.1 up to 70 GPa[11].

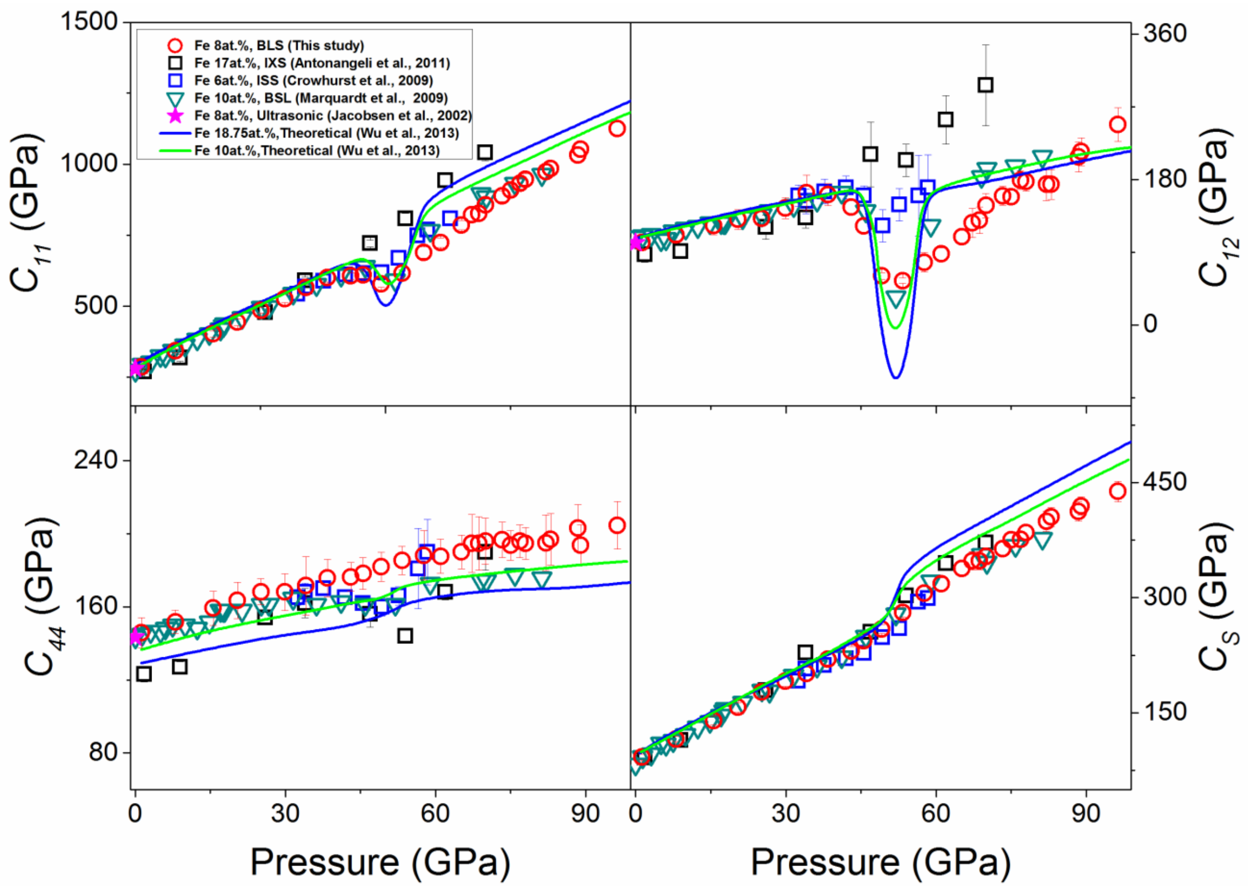

Fig. S6. Comparison of the elastic constants of ferropericlase as a function of pressure. C_S is defined as $(C_{11}-C_{12})/2$. Red circles: combined BLS and ISS measurements with 8% iron in this study; black squares: IXS study with 17% iron content[19]; green circles: BLS study below 20 GPa with 6% iron content[12]; blue triangles: ISS measurements with 6% iron[18]; dark cyan down triangles: BLS study with 10% iron[11]; magenta stars: ultrasonic measurements with 8% iron[20]; blue and green lines: theoretical results with 18.75% and 10% iron content, respectively[9].

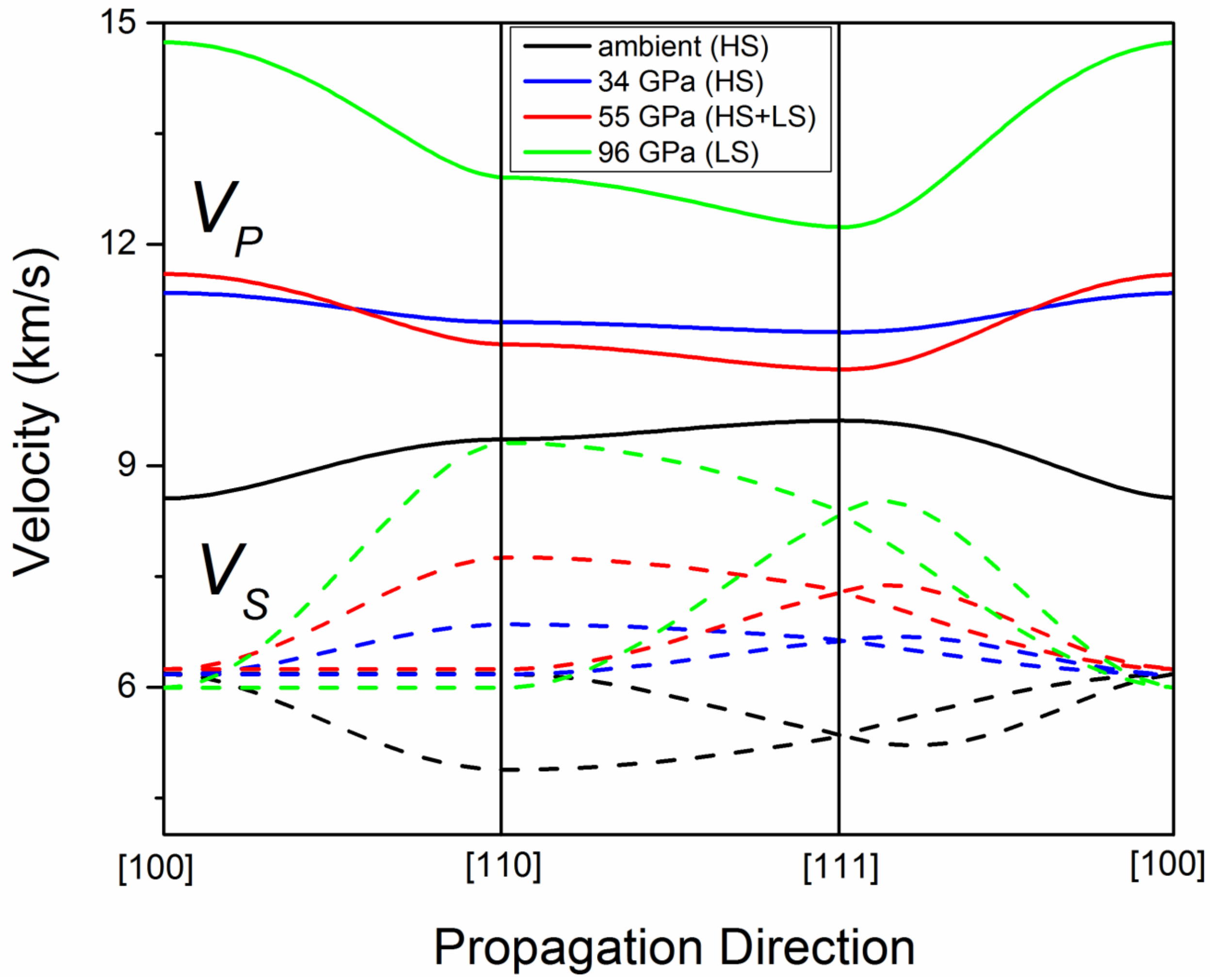

Fig. S7. Compressional (V_P) and shear (V_S) wave velocities of the single-crystal ferropericlase ($(Mg_{0.92}Fe_{0.08})O$) as a function of the propagation direction at high pressures. The velocities at representative pressures are plotted to highlight the changes in compressional and shear wave anisotropies across the spin transition.

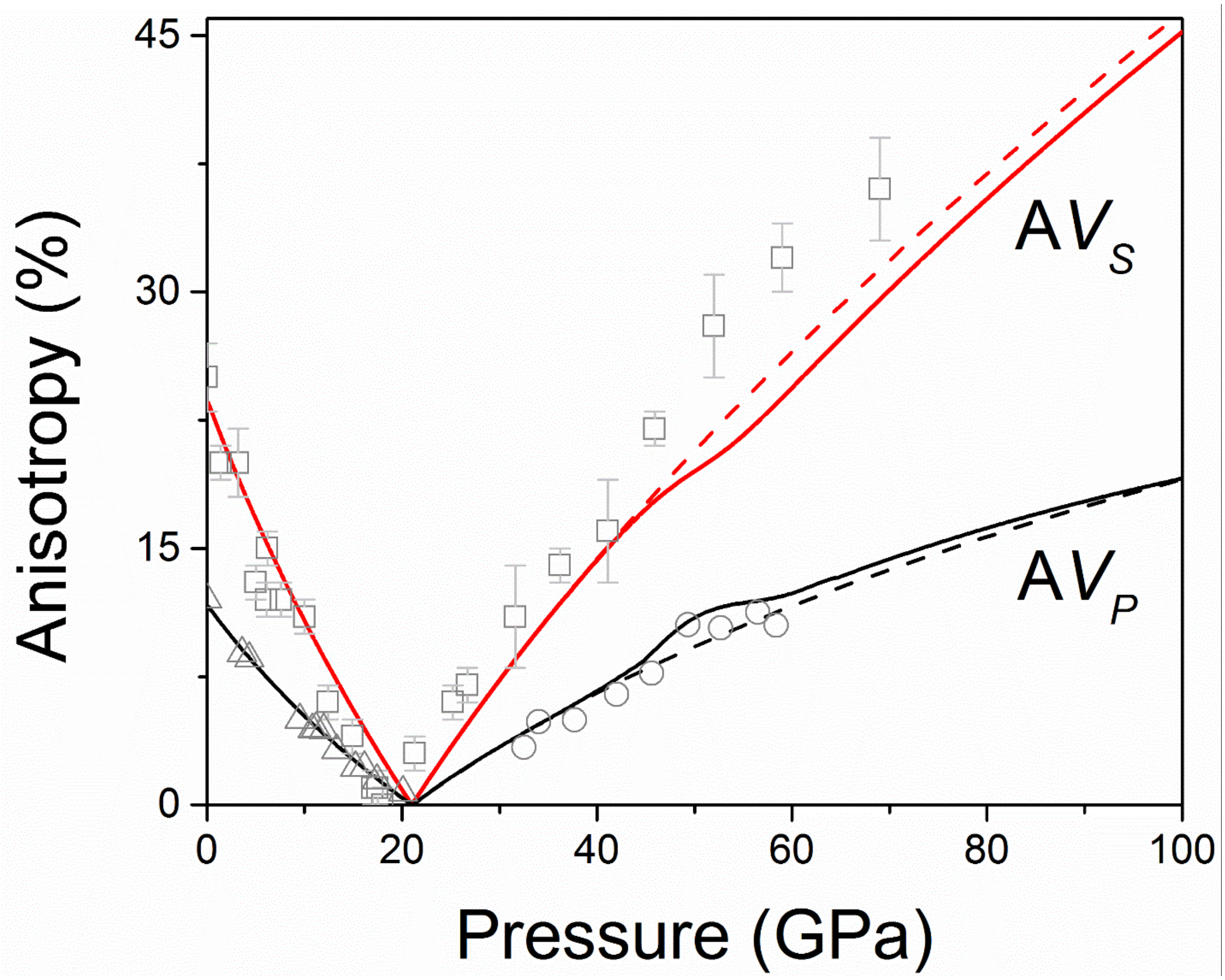

Fig. S8. Compressional and shear wave anisotropy as a function of pressure at 300 K. Red lines: shear wave anisotropy; black lines: compressional wave anisotropy. Dashed lines are the extrapolated anisotropies for the HS state that are plotted for comparison. Open squares: shear wave anisotropy calculated from directly measured velocities along [100] and [110] using equation (V_S [100] – V_S [110]) / (V_S [100] + V_S [110])/2 via BLS measurement[11]; open triangles: compressional wave anisotropy calculated from elastic constants measured by BLS using Christoffel's equations[12]; open circles: compressional wave anisotropy calculated from elastic constants measured by ISS using Christoffel's equations[18] .

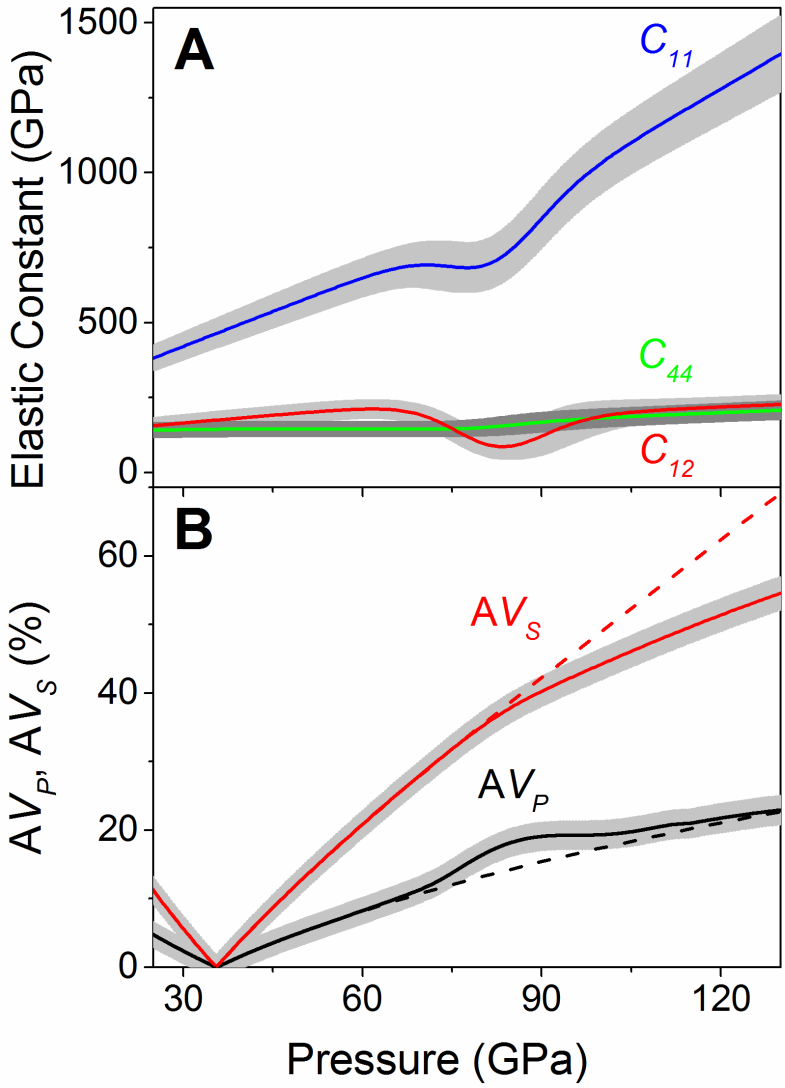

Fig. S9. Modelled elastic properties of ferropericlase $(Mg_{0.92}Fe_{0.08})O$ across the spin transition zone along an expected lower-mantle geotherm. (A) Elastic constants: C_{11}, C_{12}, and C_{44} plotted as blue, red, and green lines, respectively. (B) V_P and V_S anisotropy. Red line: V_S anisotropy; black line: V_P anisotropy. The V_P and V_S anisotropies are calculated using the equations $AV_P = (V_P max - V_P min)/2(V_P max + V_P min)$ and $AV_S = (V_S max - V_S min)/2(V_S max + V_S min)$, where subscripted *min* and *max* represent the minimum and maximum velocity of the single crystal,

respectively. Grey shaded areas show the uncertainties calculated using standard error propagations.

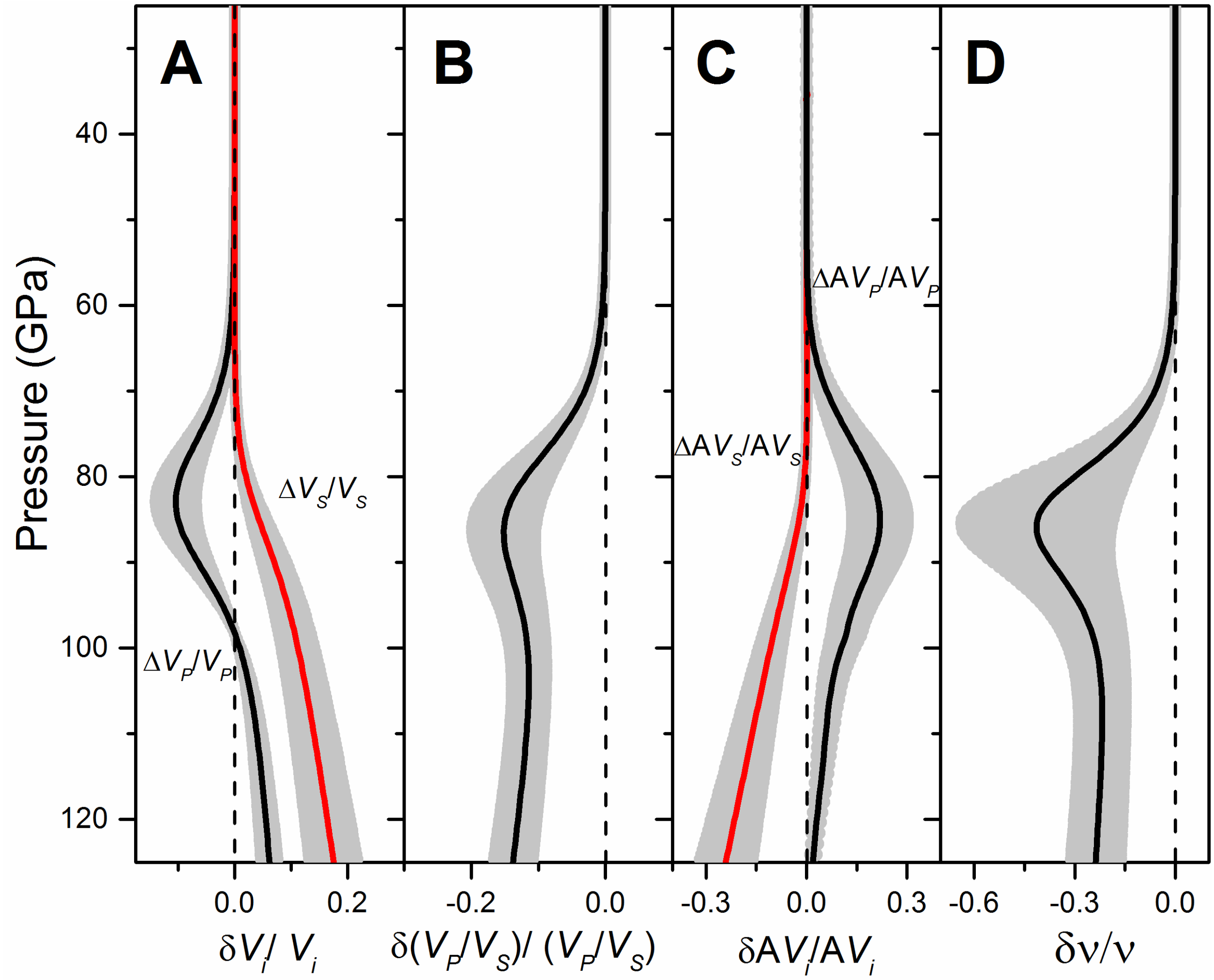

Fig. S10. Variations of the seismic parameters of ferropericlase ($(Mg_{0.92}Fe_{0.08})O$) as a function of depth along an expected lower-mantle geotherm. The variations were derived using the modelled seismic parameters of the high-spin ferropericlase as the reference. (A) Aggregate V_P and V_S; (B) V_P/V_S ratio; (C) V_P and V_S anisotropy; (D) Poisson's ratio.

Table S1. Experimental results for ferropericlase ($(Mg_{0.92}Fe_{0.08})O$). V_P velocities were derived from ISS measurements while V_S velocities were from BLS measurements.

Pressure	Density g/cm^3	V_P [100] km/s	V_P [110] km/s	V_S [100] km/s	V_S [110] km/s	C_{11} GPa	C_{12} GPa	C_{44} GPa
**Run 1**								
1.3(0.1)	3.80	8.61(0.08)	9.52(0.09)	6.14(0.07)	4.97(0.06)	287(8)	102(9)	146(8)
8.0(0.1)	3.95	9.21(0.08)	9.87(0.09)	6.12(0.07)	5.46(0.07)	343(8)	112(7)	152(6)
15.6(0.2)	4.11	9.83(0.10)	10.19(0.09)	6.17(0.07)	5.87(0.07)	403(11)	123(11)	159(9)
20.4(0.0)	4.20	10.23(0.07)	10.42(0.06)	6.19(0.10)	6.15(0.06)	445(12)	131(12)	163(10)
25.2(0.3)	4.28	10.56(0.07)	10.64(0.07)	6.19(0.10)	6.47(0.07)	486(10)	132(8)	168(7)
29.9(0.5)	4.36	10.93(0.06)	10.81(0.07)	6.15(0.10)	6.65(0.07)	527(14)	144(13)	168(10)
34.2(0.6)	4.43	11.29(0.07)	11.02(0.07)	6.21(0.10)	6.75(0.09)	566(20)	164(22)	172(16)
38.5(0.8)	4.49	11.50(0.09)	11.19(0.09)	6.19(0.10)	7.03(0.10)	601(15)	161(14)	176(10)
43.1(0.9)	4.56	11.43(0.15)	11.08(0.07)	6.12(0.22)	7.15(0.10)	607(13)	146(10)	176(8)
45.6(1.0)	4.60	11.43(0.12)	10.94(0.07)	6.15(0.20)	7.31(0.10)	610(14)	122(9)	178(9)
49.2(1.2)	4.67	11.05(0.08)	10.44(0.07)	6.16(0.20)	7.49(0.11)	579(12)	61(4)	182(8)
53.3(1.1)	4.28	11.30(0.07)	10.55(0.06)	6.17(0.20)	7.72(0.11)	616(12)	55(3)	176(8)
57.7(1.2)	4.81	11.84(0.10)	11.01(0.08)	6.13(0.10)	8.02(0.11)	689(19)	78(12)	188(14)
61.0(1.5)	4.86	12.11(0.08)	11.14(0.06)	6.12(0.15)	8.13(0.18)	724(16)	88(7)	188(9)
65.2(1.0)	4.91	12.57(0.08)	11.46(0.07)	6.14(0.15)	8.33(0.11)	786(17)	109(8)	190(9)
67.3(0.4)	4.94	12.77(0.13)	11.76(0.17)	6.14(0.11)	8.45(0.20)	823(24)	127(18)	195(16)
70.0(2.0)	4.97	13.00(0.12)	11.95(0.14)	6.16(0.11)	8.49(0.10)	856(22)	148(15)	196(13)
75.0(2.0)	5.03	13.36(0.19)	12.10(0.19)	6.11(0.11)	8.67(0.10)	909(18)	159(10)	194(8)
78.0(2.0)	5.06	13.58(0.20)	12.30(0.23)	6.10(0.11)	8.75(0.21)	947(20)	177(11)	195(9)
83.0(2.0)	5.12	13.74(0.10)	12.41(0.30)	6.06(0.11)	8.94(0.21)	985(26)	174(18)	197(14)
89.0(2.0)	5.19	14.17(0.28)	12.69(0.40)	6.03(0.11)	9.02(0.11)	1053(24)	215(17)	194(11)
**Run 2**								
68.7(1.3)	4.95	12.78(0.13)	11.76(0.07)	6.13(0.10)	8.43(0.10)	826(23)	130(17)	195(15)
73.3(1.5)	5.01	13.21(0.10)	12.07(0.17)	6.16(0.10)	8.56(0.14)	888(19)	160(12)	197(10)
76.9(1.1)	5.05	13.49(0.10)	12.28(0.20)	6.12(0.10)	8.67(0.15)	932(19)	179(12)	196(9)
82.1(1.6)	5.11	13.75(0.21)	12.30(0.30)	6.13(0.10)	8.86(0.10)	973(27)	174(20)	195(15)
88.5(1.1)	5.18	14.00(0.25)	12.70(0.20)	6.13(0.10)	8.96(0.10)	1033(25)	208(19)	203(13)
96.4(1.7)	5.27	14.50(0.28)	13.10(0.20)	6.10(0.15)	9.17(0.16)	1125(27)	248(21)	205(13)

Table S2. Elasticity of ferropericlase $(Mg_{0.92}Fe_{0.08})O$ in the high-spin and low-spin state at 300 K.

	$(\partial C_{11}/\partial P)_T$	$(\partial C_{12}/\partial P)_T$	$(\partial C_{44}/\partial P)_T$
**HS**	8.99 (0.1)	1.95(0.12)	1.22(0.10)
**LS**	9.9 (0.2)	2.2(0.2)	1.3(0.2)

Supplementary References:

1 Tsuchiya, T., Wentzcovitch, R. M., da Silva, C. R. S. & de Gironcoli, S. Spin transition in magnesiowüstite in Earth's lower mantle. *Phys. Rev. Lett.* **96**, 198501 (2006).

2 Mao, Z., Lin, J. F., Liu, J. & Prakapenka, V. B. Thermal equation of state of lower-mantle ferropericlase across the spin crossover. *Geophys. Res. Lett.* **38**, L23308 (2011).

3 Wentzcovitch, R. *et al.* Anomalous compressibility of ferropericlase throughout the iron spin cross-over. *Proc. Natl. Acad. Sci. USA* **106**, 8447 (2009).

4 Tange, Y., Nishihara, Y. & Tsuchiya, T. Unified analyses for P-V-T equation of state of MgO: A solution for pressure-scale problems in high P-T experiments. *J. Geophys. Res-Sol. Ea. (1978–2012)* **114** (2009).

5 Every, A. General closed-form expressions for acoustic waves in elastically anisotropic solids. *Phys. Rev. B* **22**, 1746 (1980).

6 Yang, J., Mao, Z., Lin, J.-F. & Prakapenka, V. B. Single-crystal elasticity of the deep-mantle magnesite at high pressure and temperature. *Earth Planet. Sci. Lett.* **392**, 292-299 (2014).

7 Birch, F. Finite strain isotherm and velocities for single-crystal and polycrystalline NaCl at high pressures and 300 K. *J. Geophys. Res.* **83**, 1257-1268 (1978).

8 Poirier, J.-P. *Introduction to the Physics of the Earth's Interior*. (Cambridge University Press, 2000).

9 Wu, Z., Justo, J. F. & Wentzcovitch, R. M. Elastic anomalies in a spin-crossover system: ferropericlase at lower mantle conditions. *Phys.Rev.Lett.* **110**, 228501 (2013).

10 Nye, J. F. *Physical properties of crystals: their representation by constants and matrices*. (Oxford university press, 1985).

11 Marquardt, H. *et al.* Elastic shear anisotropy of ferropericlase in Earth's lower mantle. *Science* **324**, 224 (2009).

12 Jackson, J. M. *et al.* Single-crystal elasticity and sound velocities of ($Mg_{0.94}Fe_{0.06}$) O ferropericlase to 20 GPa. *J. Geophys. Res.* **111** (2006).

13 Brown, J. & Shankland, T. Thermodynamic parameters in the Earth as determined from seismic profiles. *Geophys. J. Roy. Astr. S.* **66**, 579-596 (1981).

14 Lin, J. F. *et al.* Spin transition zone in Earth's lower mantle. *Science* **317**, 1740 (2007).

15 Karki, B. B., Stixrude, L. & Wentzcovitch, R. M. High-pressure elastic properties of major materials of Earth's mantle from first principles. *Rev. Geophys.* **39**, 507–534 (2001).

16 Fei, Y. *et al.* Toward an internally consistent pressure scale. *Proc. Natl. Acad. Sci. USA* **104**, 9182-9186 (2007).

17 Speziale, S. *et al.* Iron spin transition in Earth's mantle. *Proc. Natl. Acad. Sci. USA* **102**, 17918 (2005).

18 Crowhurst, J., Brown, J., Goncharov, A. & Jacobsen, S. Elasticity of (Mg,Fe)O through the spin transition of iron in the lower mantle. *Science* **319**, 451 (2008).

19 Antonangeli, D. *et al.* Spin crossover in ferropericlase at high pressure: A seismologically transparent transition? *Science* **331**, 64 (2011).

20 Jacobsen, S. D. *et al.* Structure and elasticity of single-crystal (Mg,Fe)O and a new method of generating shear waves for gigahertz ultrasonic interferometry. *J. Geophys. Res.* **107**, 2037 (2002).
